# Retarding Effect and Hydration Mechanism of Sodium Polyacrylate on Magnesium Potassium Phosphate Cement

**DOI:** 10.3390/ma19071349

**Published:** 2026-03-28

**Authors:** Yunpeng Cui, Runqing Liu, Yuanquan Yang, Bo Pang, Yihe Wang

**Affiliations:** School of Architecture and Civil Engineering, Liuzhou Institute of Technology, Liuzhou 545616, China; cuiyunpeng@sylu.edu.cn (Y.C.); 13940195514@163.com (R.L.); yangyuanquan@sylu.edu.cn (Y.Y.); pangbo0829@sylu.edu.cn (B.P.)

**Keywords:** magnesium phosphate cement, sodium polyacrylate, reaction mechanism, hydration

## Abstract

Magnesium phosphate cement (MPC) is a type of rapid-hardening inorganic cementitious material, which has important application value in rapid road repair, solidification of hazardous and radioactive waste, and other fields. However, it suffers from excessively fast setting and hardening and a short working time retention, which severely restrict its engineering application. Therefore, the development of high-efficiency set retarders is of great significance for optimizing MPC performance, enhancing its construction workability, and expanding its application scope. In this study, the effect of sodium polyacrylate (PAAS) on the setting and hardening of magnesium potassium phosphate cement (MKPC) was investigated by testing the setting time and fluidity at a low water-to-solid ratio (W/S = 0.18). Through pH and electrical conductivity measurements, combined with XRD, TG/DTG, and FTIR characterizations, we elucidated the retarding mechanism of PAAS on MKPC using a high water-to-solid ratio (W/S = 10). The results indicate that the setting time of MKPC is positively correlated with the PAAS dosage, whereas the fluidity and compressive strength exhibited a negative correlation with the PAAS dosage. Additionally, PAAS reduces the total heat release and the heat release rate of MKPC. The addition of PAAS increased the pH of the suspension, thereby reducing the solubility of MgO, but did not inhibit the dissolution of KH_2_PO_4_. The carboxylate groups in PAAS chemically reacted with Mg^2+^ on the surface of MgO to form magnesium carboxylate complexes (Mg-PAA), which remained as precipitates in the MKPC suspension system, thus reducing the amount of available Mg^2+^ participating in the hydration reaction. Furthermore, PAAS had no effect on the final precipitate composition at the end of hydration, which was composed of MgKPO_4_·6H_2_O and Mg_3_(PO_4_)_2_·22H_2_O in all cases.

## 1. Introduction

Magnesium phosphate cement (MPC) is a new type of inorganic cementitious material characterized by significantly improved early strength [1,2,3], fast initial hardening [2,3,4], good bonding performance, small volume shrinkage [3,5,6], excellent high-temperature resistance [7,8], and outstanding frost–thaw cycling resistance [9,10]. It shows great potential application value in engineering projects such as infrastructure repair [11,12,13,14,15,16,17], structural reinforcement [8,18,19,20,21], and emergency repair [22,23].

MPC is mainly prepared by mixing acid-soluble phosphates (${\mathrm{KH}}_{2}{\mathrm{PO}}_{4}$, ${\mathrm{NH}}_{4}H_{2}{\mathrm{PO}}_{4}$) with dead-burned MgO to undergo an exothermic acid–base neutralization reaction. When ${\mathrm{NH}}_{4}H_{2}{\mathrm{PO}}_{4}$ is used to prepare MPC, the main hydration product is ${\mathrm{MgNH}}_{4}{\mathrm{PO}}_{4}\cdot 6H_{2}O$ (Equation (1)) [24,25]. However, toxic ammonia gas is released during the setting and hardening process, thus limiting its application. Usually, ${\mathrm{KH}}_{2}{\mathrm{PO}}_{4}$ is used to replace ${\mathrm{NH}}_{4}H_{2}{\mathrm{PO}}_{4}$ for the preparation of magnesium potassium phosphate cement (MKPC) [26,27,28,29], and the main hydrated precipitate is ${\mathrm{MgKPO}}_{4}\cdot 6H_{2}O$ [29,30], commonly known as potassium struvite (Equation (2)).

(1) $$MgO+{NH_{4}H}_{2}{PO}_{4}+5H_{2}O\to {Mg{NH}_{4}PO}_{4}\cdot {6H}_{2}O$$

(2) $$MgO+{KH}_{2}{PO}_{4}+5H_{2}O\to {MgKPO}_{4}\cdot {6H}_{2}O$$

The hydration mechanism of MKPC is a complex process involving MgO and ${\mathrm{KH}}_{2}{\mathrm{PO}}_{4}$. Its hydration process and hydration products are related to the magnesium-to-phosphorus ratio (M/P), water-to-solid ratio (W/S), set retarders, temperature, and other factors. Under different conditions, the hydration process of MKPC produces various hydration products, such as ${\mathrm{MgKPO}}_{4}\cdot 6H_{2}O$ (K-struvite), ${\mathrm{MgHPO}}_{4}\cdot 7H_{2}O$ (Phosphorrösslerite), ${\mathrm{Mg}}_{3}{\left({\mathrm{PO}}_{4}\right)}_{2}\cdot 22H_{2}O$ (Cattiite), ${\mathrm{MgHPO}}_{4}\cdot 3H_{2}O$ (Newberyite), etc. A large number of studies have shown that the reaction mechanism of MKPC involves an exothermic acid–base neutralization reaction between dead-burned MgO and acid-soluble phosphates [31,32]. This reaction proceeds rapidly and generates a high-strength hydration product within minutes. The exothermic process further increases the temperature of the paste, thereby producing an autocatalytic effect, leading to almost instantaneous setting and hardening of the paste [25]. Therefore, modification treatment of MKPC is required. Usually, set retarders are added to improve the workability of MKPC and extend its hydration time. Lahalle et al. [25] investigated the effect of boric acid on the hydration process of MPC in a highly diluted MKPC suspension, indicating that the hydration of MKPC is a dissolution-precipitation process. Boric acid slows down the formation of MPC hydrates mainly through two ways: (1) stabilizing cations (Mg^2+^, K^+^) in the solution to offset the negative charge formed by borates at pH higher than 6; (2) initially precipitating minerals containing borates and phosphates; it may generate pH and concentration conditions to slow down the precipitation of hydrates. Wei et al. [33] studied the effect of composite set retarders of boric acid and triethanolamine (TEA) on the early hydration performance of MKPC, showing that TEA inhibits the early hydration reaction of MKPC, hinders the dissolution of MgO, forms complexes with K^+^, and inhibits the formation of ${\mathrm{MgKPO}}_{4}\cdot 6H_{2}O$ without affecting the types of hydration products. Coumes et al. [34] studied the effect of aluminum nitrate on the hydration process of MKPC in a diluted suspension, and found that aluminum nitrate reduced the pH of the suspension, inhibited the precipitation of K-containing magnesium phosphate hydrates (${\mathrm{Mg}}_{2}{\mathrm{KH}({\mathrm{PO}}_{4})}_{2}\cdot 15H_{2}O$, ${\mathrm{MgKPO}}_{4}\cdot 6H_{2}O$), and delayed the precipitation of ${\mathrm{Mg}}_{3}{\left({\mathrm{PO}}_{4}\right)}_{2}\cdot 22H_{2}O$ and ${\mathrm{MgHPO}}_{4}\cdot 3H_{2}O$. Gelli et al. [31] used borax as a retarder to study its effect on the setting and hardening of MPC. The study did not find complexes between borax and Mg^2+^; instead, the addition of borax promoted the precipitation of struvite and reduced the precipitation of ${\mathrm{MgHPO}}_{4}\cdot 3H_{2}O$. Xu et al. [35] studied the effect of a high M/P ratio on the hydration process of magnesium phosphate cement under conditions of highly diluted MKPC suspensions. The results showed that a high M/P ratio could inhibit the formation of hydrates such as potassium-free magnesium phosphate (MgHPO_4_·3H_2_O), while mainly forming MgKPO_4_·6H_2_O.

In summary, the use of different sets of retarders has different effects on the performance and hydration mechanism of MPC. The most commonly used ones are NaB_4_O_7_·10H_2_O, H_3_BO_3_ [21,25,36,37]. However, boron is prone to precipitation under certain conditions and exhibits reproductive toxicity [4,38,39], which may pose hazards to the environment and human health. Therefore, selecting an appropriate set of retarders is crucial for expanding the application range of MKPC. PAAS, a superabsorbent resin, has the advantages of good biodegradability, non-toxicity [40], and environmental friendliness. It is widely used in agriculture, medicine, construction, and other fields. In cement concrete, PAAS is often used as an internal curing admixture to improve the performance of cement-based materials [41,42]. In this study, the effect of PAAS on the setting and hardening of MKPC was investigated by testing the setting time and fluidity at a low water-to-solid ratio (W/S = 0.18). Furthermore, the retarding mechanism of PAAS on MKPC was studied at a high water-to-solid ratio (W/S = 10) by measuring the pH value and electrical conductivity, combined with characterizations including XRD, TG/DTG, and FTIR, providing new insights for future theoretical research and engineering applications of MKPC.

## 2. Materials and Methods

### 2.1. Materials

**Figure 1:**
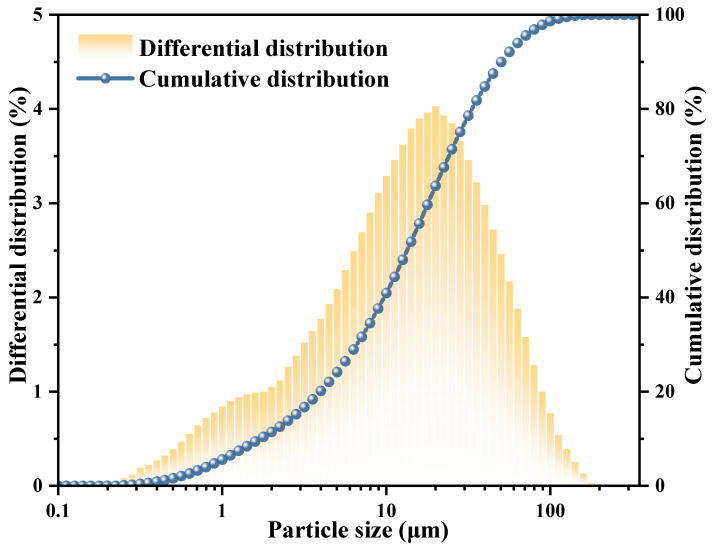
Particle size distribution of MgO.

Dead-burned MgO was prepared via high-temperature calcination of magnesite at 1600–1800 °C, followed by grinding to a uniform particle size. Its reaction activity was evaluated by the acetic acid method [43], and the obtained reaction time was 236 min. The specific surface area of MgO was 757 m^2^/kg, and the characteristic values of particle size distribution were D10 = 1.903 μm, D50 = 15.03 μm, and D90 = 56.41 μm. The particle size distribution is shown in Figure 1, and the chemical composition is shown in Table 1. Sodium polyacrylate ((C_3_H_3_NaO_2_)_n_, referred to as PAAS) has a weakly alkaline aqueous solution. KH_2_PO_4_ (purity > 99%) and PAAS were both purchased from Tianjin Damao Chemical Reagent Factory in China. Deionized water was used for all test water.

### 2.2. Mix Design

In this experiment, the molecular molar ratio (M/P) of dead-burned MgO to ${\mathrm{KH}}_{2}{\mathrm{PO}}_{4}$ was 8/1. A low water-to-solid ratio (W/S) of 0.18 was adopted in the setting, and hardening tests, and the PAAS dosage was 0%, 4%, 8%, 12%, and 16% of the total mass of MgO and ${\mathrm{KH}}_{2}{\mathrm{PO}}_{4}$. The specific mix ratio design is shown in Table 2. For the study of the retarding mechanism, a high W/S of 10 was used, and the PAAS dosage was 0%, 4%, and 16% of the deionized water mass. The specific mix ratio design is shown in Table 3.

### 2.3. Test Methods

(1) Work performance.

The setting time and fluidity were measured in accordance with the method specified in JC/T2537-2019 [44] “Magnesium Phosphate Repair Mortar”. Among them, the setting time was tested using a Vicat apparatus prepared by New Era Testing Instruments Co., Ltd. in Shenyang, Liaoning, China. Due to the short interval between the initial setting time and the final setting time, only the final setting time was measured in the experiment.

(2) Mechanical properties.

In the experiment, a YAW-300D type pressure testing machine produced by New Era Testing Instruments Co., Ltd. in Shenyang, Liaoning, China, was used to determine the compressive strength of MKPC specimens at 3 h, 3 d, and 28 d, respectively, in accordance with the method specified in JC/T2537-2019 “Magnesium Phosphate Repair Mortar”.

(3) Heat of hydration.

The TAM-air instrument produced by New Era Testing Instruments Co., Ltd. in Shenyang, Liaoning, China, was adopted, with the temperature set at 20 °C. After the instrument stabilized, all the mixing water in the syringe was pushed into the ampoule containing the mixed dry materials, and the built-in stirrer was started to stir in the bottle for 2 min, while data collection was initiated simultaneously.

(4) Determination of pH and conductivity.

**Figure 2:**
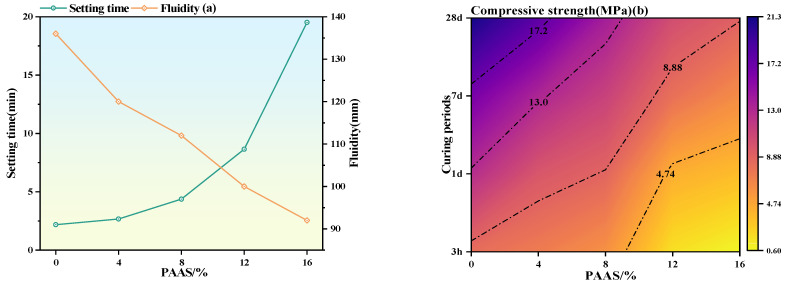
MKPC working performance (**a**) and compressive strength (**b**) with different PAAS contents.

The test used an S470-K bench-top pH/conductivity meter produced in by New Era Testing Instruments Co., Ltd. Shenyang, Liaoning, China, the experiment was conducted under mechanical stirring at 25 °C. Before each measurement, the pH and conductivity were calibrated using standard buffer solutions with pH values of 4.01, 7, and 9.21, and a conductivity standard solution of 1408 μS/cm. First, ${\mathrm{KH}}_{2}{\mathrm{PO}}_{4}$ and PAAS were placed in a 500 mL beaker containing deionized water, and stirring was conducted in a 25 °C thermostatic water bath (25 g of ${\mathrm{KH}}_{2}{\mathrm{PO}}_{4}$ can be dissolved in 100 mL of water at 25 °C). The endothermic dissolution of ${\mathrm{KH}}_{2}{\mathrm{PO}}_{4}$ caused the temperature of the suspension to decrease. When the temperature reached 25 °C again, dead-burned MgO was added, and the test was started simultaneously. The pH and conductivity data of MKPC at hydration times of 12 h and 24 h were continuously recorded, respectively. Figure 2 shows the simplified model of the pH/conductivity integrated tester.

(5) Inductively Coupled Plasma Optical Emission Spectrometry (ICP-OES).

The concentrations of Mg, K, P, and Na ions were determined using an Inductively Coupled Plasma Optical Emission Spectrometer (ICP-OES, Avio200) produced by Thermo Fisher Scientific Co., Ltd. in Shanghai, China. Samples were diluted with 2% dilute nitric acid before measuring the concentrations of different ions. During the measurement, standard solutions of Mg, K, P, and Na with concentrations of 20, 40, 60, and 80 μg/mL were used as calibrators. Samples were extracted at different hydration times (determined by pH and conductivity data) during the solution hydration process, and the ion concentrations at these specific hydration times were measured.

(6) XRD.

The precipitates were analyzed qualitatively and quantitatively using a Rigaku Ultima IV X-ray diffraction (XRD) analyzer produced by Thermo Fisher Scientific Co., Ltd. in Shanghai, China, with a scanning range of 5–65° and a scanning speed of 6°/min. Samples were collected at different hydration times (determined by pH and conductivity data) during the solution hydration process. Then the precipitates were separated from the filtrate using a filter membrane. The precipitates were washed with absolute ethanol, filtered, dried, and then subjected to qualitative and quantitative analysis by XRD. For quantitative analysis, 20% ZnO was added as an internal standard sample, and absolute ethanol was used as the medium to homogenously mix the sample and the internal standard. Finally, quantitative phase analysis was performed using Topas3 combined with the Rietveld method [45].

(7) FTIR.

The vibrational and rotational modes of different molecular bonds in samples at different hydration times (determined by pH and conductivity data) were detected using a Nicolet iS50 Fourier Transform Infrared (FT-IR) spectrometer produced by Thermo Fisher Scientific Co., Ltd. in Shanghai, China, with a scanning wavenumber range of 4000–400 ${\mathrm{cm}}^{-1}$.

(8) TG/DTG.

Samples at different hydration times (hydration times determined by pH and conductivity data) were analyzed using a STA449 synchronous thermal analyzer produced by Thermo Fisher Scientific Co., Ltd. in Shanghai, China. The samples were heated from 25 °C to 900 °C at a heating rate of 10 °C/min under a nitrogen atmosphere.

(9) SEM.

The apparent morphology of samples at different hydration times (hydration times determined by pH and conductivity data) was observed using a Hitachi S-3400N scanning electron microscope produced by Thermo Fisher Scientific Co., Ltd. in Shanghai. Samples were prepared using the same method as for XRD qualitative analysis and sputter-coated with gold before observation.

## 3. Results

### 3.1. Macro Performance Testing

Figure 2 shows the workability (a) and compressive strength (b) of MKPC with different PAAS dosages. As the PAAS dosage increased, the setting time of MKPC increased from 1.97 min to 19.52 min, representing a 795.41% increase, indicating a positive correlation between PAAS dosage and setting time. The fluidity of MKPC was negatively correlated with the PAAS dosage; as the PAAS dosage increased, the fluidity of MKPC decreased from 136 mm to 92 mm, a decrease of 32.35%. PAAS is a polymer with a large number of carboxyl (-COOH) groups on its molecular chain. When dissolved in water, PAAS can form hydrogen bonds between its carboxyl groups and water molecules. Through the action of hydrogen bonds, PAAS molecules can combine with a large number of water molecules, fixing the water molecules around their molecular chains and reducing the freely movable water, thereby decreasing the fluidity. The compressive strength of MKPC was positively correlated with the curing age and negatively correlated with the PAAS dosage. When the PAAS dosage was 16%, the compressive strength of MKPC cured for 3 h was only 0.6 MPa. When the PAAS dosage was further increased, MKPC lost its strength after 3 h of hydration and could not be applied normally.

### 3.2. Hydration Heat Test

**Figure 3:**
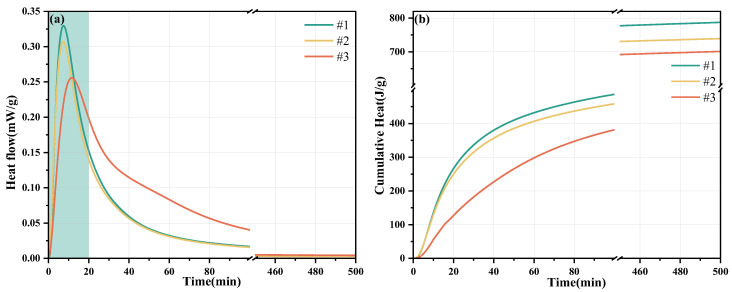
Heat release rate of MKPC with PAAS content of 0%, 4%, and 16% (**a**); total heat release (**b**).

Figure 3 presents the hydration heat release rate (a) and total heat release (b) of MKPC with PAAS dosages of 0%, 4%, and 16%. The MKPC reaction mechanism is an exothermic acid–base neutralization reaction, and the heat released increases the temperature of the paste, leading to further acceleration of the hydration reaction. The main exothermic reaction of MKPC occurs within the first 80 min; as the hydration process proceeds, the hydration heat release rate gradually decreases, the total heat release tends to stabilize, and the main exothermic reaction ends. The exothermic peak in the first 20 min is affected by various factors, including the endothermic dissolution of ${\mathrm{KH}}_{2}{\mathrm{PO}}_{4}$, the dissolution of MgO, and the formation of amorphous hydration products and K-struvite. With an increasing PAAS dosage, the peak area of the Exothermic Peak decreases, the peak shape becomes wider, and the peak position shifts to the right. This indicates that PAAS prolongs the formation time of amorphous hydration products, delays the formation time of K-struvite, and reduces the total heat release of MKPC. When the PAAS dosage is 0%, the total heat release of MKPC is 779 J/g; when the PAAS dosage is 16%, the total heat release of MKPC is 694 J/g, a decrease of 10.91%. The hydration reaction of MKPC is not complete, and the total heat release cannot be directly measured through experiments. According to Equation 1, the theoretical total heat release of MKPC, Q_∞_, is estimated to be 780 J/g, 730 J/g, and 700 J/g, respectively. Subsequently, the degree of heat release of MKPC at different hydration times is calculated according to Equation (2). When the hydration reaction reaches 80 min, the degree of hydration of M-0 reaches 61.5%, while that of M-16 is only 45.7%. With the increasing PAAS content, the hydration heat release rate, total heat released, and degree of hydration of MKPC all decrease.

(3) $$\frac{1}{Q}=\frac{1}{Q_{\infty }}+\frac{t_{50}}{Q_{\infty }\left(t-t_{0}\right)}$$

(4) α=QQ∞

*Q*: Cumulative heat release at time t,

*Q*_∞_: Final heat release,

*t*_50_: Time to reach a heat release of *Q*_∞_,

*t*_0_: Hydration reaction start time,

*α*: Degree of hydration.

### 3.3. pH and Conductivity

**Figure 4:**
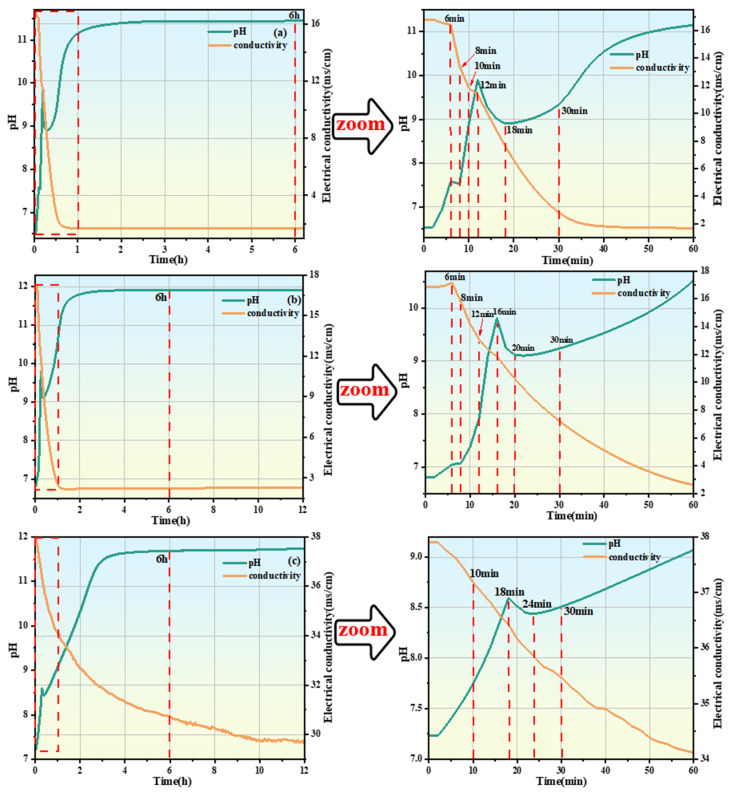
pH and conductivity curves of MKPC suspension at different hydration times: (**a**) #1 MKPC; (**b**) #2 MKPC; (**c**) #3 MKPC.

Figure 4 shows the pH and conductivity curves of the #1, #2, and #3 MKPC suspensions at hydration times of 6 h, 12 h, and 60 min, indicating the hydration times of the extracted samples. The red line represents the sampling time of solid and liquid samples of the suspension. The determination of pH and conductivity was carried out under a constant temperature water bath at 25 °C. ${\mathrm{KH}}_{2}{\mathrm{PO}}_{4}$ fully dissolved to release OH^−^ (Equations (3) and (4)), so the pH of the #1 suspension was weakly acidic at 0 min. The #2 and #3 suspensions with added PAAS underwent hydrolysis reactions (Equation (5)), producing OH^−^ to neutralize part of the OH^−^ generated by the dissolution of ${\mathrm{KH}}_{2}{\mathrm{PO}}_{4}$ in water. In addition, Na^+^ was introduced into the suspensions, resulting in higher conductivity and pH of the #2 and #3 suspensions than those of the #1 suspension at 0 min. The #2 suspension was still weakly acidic overall, while the #3 suspension was weakly alkaline. After ${\mathrm{KH}}_{2}{\mathrm{PO}}_{4}$ was fully dissolved, MgO was added to release ${\mathrm{Mg}}^{2+}$ and OH^−^ (Equation (6)).

At 0 min, with the increase of PAAS dosage, the pH and conductivity of the #1, #2, and #3 MKPC suspensions gradually increased, which prolonged the time for the pH of the suspension to reach an obvious maximum value. Moreover, the initial slope of the pH-time curve of the #2 and #3 suspensions was lower than that of the #1 MKPC suspension, indicating that the increase in pH of the PAAS-containing suspension led to a decrease in OH^−^, thereby inhibiting the dissolution of MgO. For the #1 MKPC suspension, the pH gradually increased while the conductivity slightly decreased within the first 6 min of hydration, indicating that the formation of precipitates and the dissolution of MgO proceeded simultaneously at this stage, and the rate of ion precipitation was greater than the rate of ion release. In contrast, for the #2 MKPC suspension, both pH and conductivity increased within the first 6 min, indicating that the rate of ion release was greater than the rate of ion precipitation or no hydration product precipitation was generated. Two obvious maximum values of pH were observed for the #1 suspension at 6 min and 12 min, corresponding to two inflection points of conductivity, indicating changes in the rates of precipitate formation and dissolution as well as changes in the types of precipitates. During this stage (6 min–12 min), the conductivity began to decrease nonlinearly, indicating that multiple precipitation reactions occurred in the suspension, while the pH decreased from 7.58 to 7.52 and then gradually increased to 9.9. In contrast, only one obvious maximum value was observed for the #2 and #3 suspensions at 16 min and 18 min, respectively. After 12 min, a short plateau period of pH was observed for the #1 suspension, followed by a gradual increase in pH to 11.2, while the conductivity continued to decrease and stabilized after 1 h. With the increase of PAAS dosage, the pH and conductivity of the #2 and #3 MKPC suspensions stabilized after 2 h and 3 h, respectively, indicating the end of the main hydration reaction.

(5) $${KH}_{2}{PO}_{4}\to K^{+}+H_{2}{PO}_{4}^{-}$$

(6) $$H_{2}{PO}_{4}^{-}\to H^{+}+{HPO}_{4}^{2-}$$

(7) $${Na}_{n}\left[PAA\right]+nH_{2}O\to \left[PAAH\right]+n{Na}^{+}+n{OH}^{-}$$

(8) $$MgO+H^{+}\to {Mg}^{2+}+{OH}^{-}$$

### 3.4. XRD

**Figure 5:**
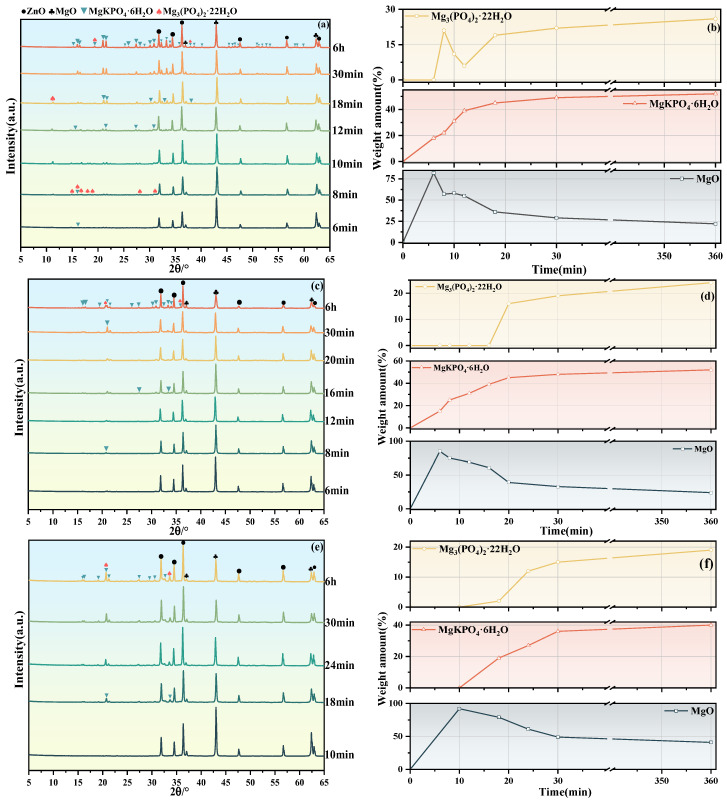
#1, #2, and #3 XRD patterns of MKPC at different hydration times qualitative/quantitative analysis: (**a**,**c**,**e**) qualitative analysis; (**b**,**d**,**f**) quantitative analysis.

Figure 5 presents the qualitative analysis of XRD patterns (a), (c), and (e) and the quantitative analysis of each substance (b), (d), and (f) of the #1, #2, and #3 MKPC suspensions at different hydration times. PAAS is a high molecular material whose molecular chains have high flexibility and amorphous characteristics. It mainly exists in an amorphous or semicrystalline state with low crystallinity and cannot be detected by XRD (Figure 5c,e). With the progress of hydration reaction for the #1, #2, and #3 MKPC, the content of MgO gradually decreased, and no ${\mathrm{KH}}_{2}{\mathrm{PO}}_{4}$ was detected, which proved that ${\mathrm{KH}}_{2}{\mathrm{PO}}_{4}$ was fully dissolved. At 6 h, the precipitate consists of MgKPO_4_·6H_2_O, Mg_3_(PO_4_)_2_·22H_2_O, and unreacted MgO. With the increase of PAAS content, the amount of MgKPO_4_·6H_2_O decreases from 52% to 40%, the amount of Mg_3_(PO_4_)_2_·22H_2_O decreases from 26% to 19%, while the amount of MgO increases from 22% to 41%.

For suspension #1, at 6 min, only one precipitate MgKPO_4_·6H_2_O was observed with a content of 18%. When hydration proceeded to 8 min, the hydration products included 21% Mg_3_(PO_4_)_2_·22H_2_O and 22% MgKPO_4_·6H_2_O. As hydration reached 6 h, the content of the precipitate MgKPO_4_·6H_2_O gradually increased to 52%, while Mg_3_(PO_4_)2·22H_2_O gradually decomposed and its content decreased. At 12 min, only a small amount of Mg_3_(PO_4_)_2_·22H_2_O was observed with a content of 6%, then its content gradually increased to 26%. For the #2 MKPC suspension, at 6 min, a small amount of 15% MgKPO_4_·6H_2_O precipitate was observed, and its content gradually increased as hydration proceeded, reaching 39% at 12 min. At 12 min, only one precipitate MgKPO_4_·6 H_2_O was detected. When hydration proceeded to 20 min, the formation of Mg_3_(PO_4_)_2_·22H_2_O was observed, with the content of both gradually increasing, reaching 52% and 24%, respectively, at 6 h. The #3 MKPC paste did not show significant hydration products at 10 min; the main process was the dissolution of MgO, with a content decrease of 8%. The first hydration product MgKPO_4_·6H_2_O appeared at 18 min with a content of 19%, and a small amount of Mg_3_(PO_4_)_2_·22H_2_O was also detected at 2%. At the same time, compared with #1 and #2 suspensions, the content was significantly lower. As hydration proceeded, the contents of both gradually increased, but remained significantly lower than those in the #1 and #2 pastes. At 6 h, the precipitates of the #1, #2, and #3 pastes were all composed of MgKPO_4_·6H_2_O, Mg_3_(PO_4_)_2_·22H_2_O, and MgO. Throughout the hydration process, no Na^+^-containing hydration products were observed, indicating that PAAS did not undergo hydration reactions and only acted to inhibit the formation of hydration products.

### 3.5. Evolution of Ion Concentrations

**Figure 6:**
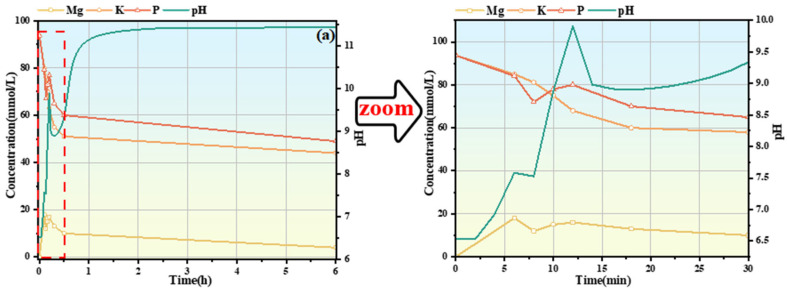

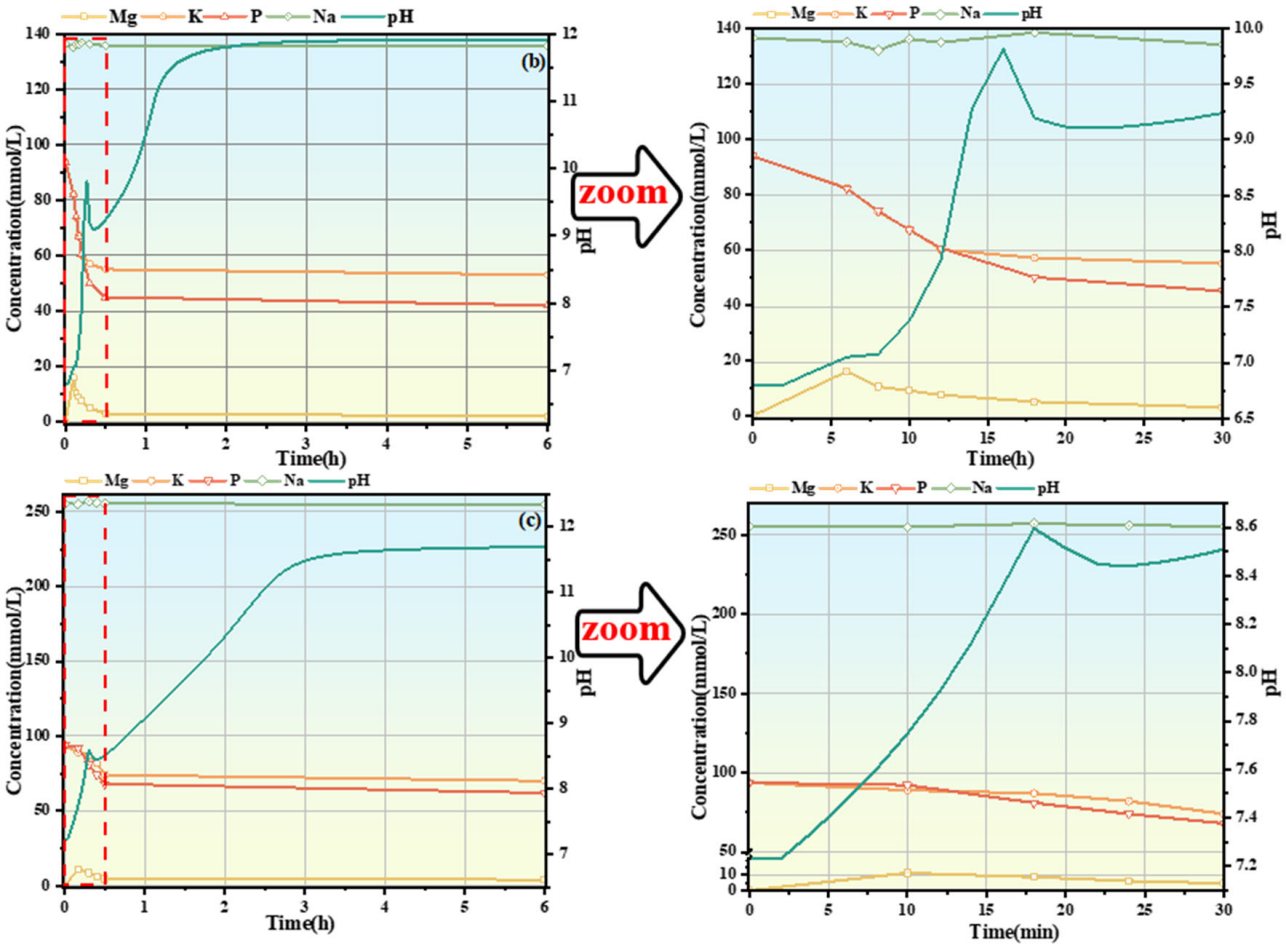
Ion concentrations (Mg, K, P, Na) of MKPC suspensions at different hydration times: (**a**) #1 MKPC; (**b**) #2 MKPC; (**c**) #3 MKPC.

Figure 6 shows the changes in Mg, K, P, and Na ion concentrations of MKPC suspensions at different hydration times: (a) #1 MKPC; (b) #2 MKPC; (c) #3 MKPC, as well as the pH curves. The red dashed area corresponds to the enlarged image. For the #1, #2, and #3 suspensions, the concentrations of each ion changed slightly after 30 min of hydration, indicating that the main precipitation of MKPC suspensions occurred within the first 30 min of hydration. During the hydration process, the concentrations of K^+^ and ${PO}_{4}^{3-}$ were always higher than that of Mg^2+^. The Na+ concentrations of the #2 and #3 suspensions remained constant throughout, and were similar to the concentrations of K^+^ and ${PO}_{4}^{3-}$ in #1 suspension at 0 min, but the ${\mathrm{Mg}}^{2+}$ concentration gradually decreased with the increase of PAAS dosage. PAAS did not participate in the hydration reaction of MKPC; instead, it increased the pH of the suspension to inhibit the dissolution of MgO, or PAAS underwent a hydrolysis reaction (Equation (5)) to generate PAA, which formed insoluble Mg-PAA precipitates with Mg^2+^ (Equation (7)), while having no effect on the dissolution of ${\mathrm{KH}}_{2}{\mathrm{PO}}_{4}$. Since the Mg^2+^ concentration is the result of the dynamic interaction between Mg^2+^ precipitation (hydration product precipitation) and release (MgO dissolution and hydration product decomposition), the changes caused by different precipitations were reflected by the changes in the concentrations of potassium and ${PO}_{4}^{3-}$.

With the addition of MgO, the hydration reaction of MKPC started. For the #1 suspension, the concentrations of K^+^ and ${PO}_{4}^{3-}$ decreased at 6 min with a consistent downward trend; combined with the XRD qualitative analysis (Figure 5a), the hydration product at this time was ${\mathrm{MgKPO}}_{4}\cdot 6H_{2}O$. The Mg^2+^ concentration continued to increase, indicating that the Mg^2+^ concentration was dominated by MgO dissolution before 6 min, while after 6 min, the Mg^2+^ concentration decreased, dominated by hydration product precipitation. At 8 min, the concentrations of K^+^ and ${PO}_{4}^{3-}$ continued to decrease, and the ${PO}_{4}^{3-}$ concentration decreased more significantly due to the formation of ${\mathrm{Mg}}_{3}{\left({\mathrm{PO}}_{4}\right)}_{2}\cdot 22H_{2}O$ precipitates. From 6 min to 12 min, the concentrations of Mg^2+^ and ${PO}_{4}^{3-}$ increased slightly, while the $K^{+}$ concentration continued to decrease. Combined with the XRD quantitative analysis (Figure 5b), the content of ${\mathrm{Mg}}_{3}{\left({\mathrm{PO}}_{4}\right)}_{2}\cdot 22H_{2}O$ gradually decreased and the content of ${\mathrm{MgKPO}}_{4}\cdot 6H_{2}O$ increased during this stage. This indicates that the ${\mathrm{Mg}}_{3}{\left({\mathrm{PO}}_{4}\right)}_{2}\cdot 22H_{2}O$ precipitates gradually dissolved to release ${\mathrm{Mg}}^{2+}$ and ${PO}_{4}^{3-}$, which then combined with K^+^ to form ${\mathrm{MgKPO}}_{4}\cdot 6H_{2}O$. Subsequently, ${\mathrm{Mg}}_{3}{\left({\mathrm{PO}}_{4}\right)}_{2}\cdot 22H_{2}O$ and ${\mathrm{MgKPO}}_{4}\cdot 6H_{2}O$ continued to form in the suspension, and the concentrations of the three ions continued to decrease.

**Figure 7:**
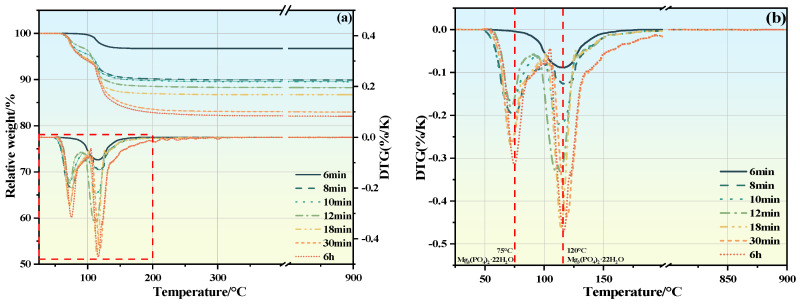

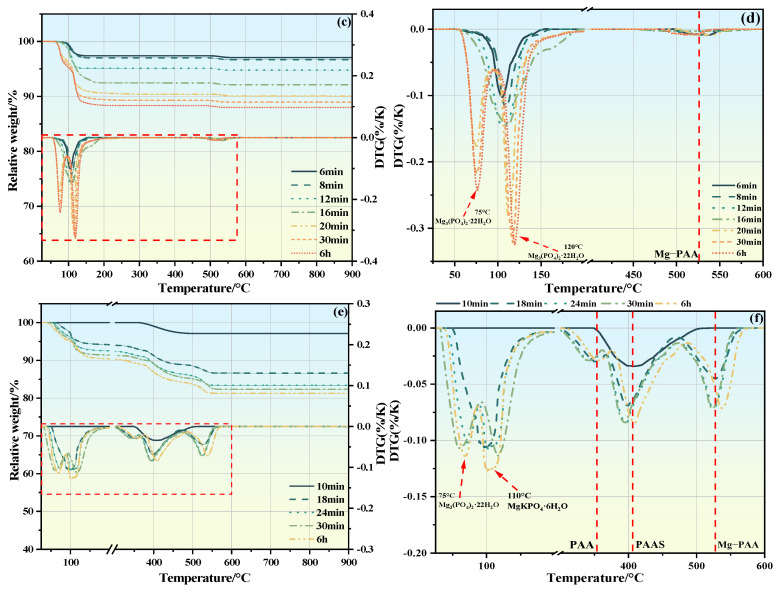
TG/DTG curves of MKPC suspension at different hydration times: TG/DTG curves of the #1 (**a**,**b**); TG/DTG curves of #2 (**c**,**d**); TG/DTG curves of #3 (**e**,**f**).

The addition of PAAS increased the pH of the suspension, inhibiting the dissolution of MgO and leading to a decrease in Mg^2+^ concentrations in #2 and #3 suspensions. For the #2 suspension, the concentrations of K^+^ and ${PO}_{4}^{3-}$ showed a consistent downward trend within the first 12 min of hydration; combined with XRD analysis (Figure 5b), ${\mathrm{MgKPO}}_{4}\cdot 6H_{2}O$ was continuously generated during this stage. Subsequently, the concentrations of K^+^ and ${PO}_{4}^{3-}$ continued to decrease, and the ${PO}_{4}^{3-}$ concentration decreased more significantly due to the formation of ${\mathrm{Mg}}_{3}{\left({\mathrm{PO}}_{4}\right)}_{2}\cdot 22H_{2}O$. The Mg^2+^ concentration continued to increase within the first 6 min of hydration and then gradually decreased. For the #3 suspension, at 0 min, the weakly alkaline pH environment further reduced the solubility of MgO. Within the first 10 min of hydration, the main process was the dissolution of MgO, with the Mg^2+^ concentration increasing, the ${PO}_{4}^{3-}$ concentration remaining unchanged, and the K^+^ concentration decreasing slightly, which was consistent with the ion concentration change rule of the #3 suspension in the initial hydration stage (10 min). In addition, according to XRD analysis (Figure 5e), no obvious hydration products were observed at 10 min, so it is speculated that PAAS underwent a hydrolysis reaction (Equation (6)) to generate PAA, which combined with K^+^ to form K-PAA (Equation (8)). No weight loss peak of K-PAA was found according to DTG analysis (Figure 7f), so the combination of K^+^ and PAA was followed by decomposition. Subsequently, the concentrations of Mg^2+^, K^+^, and ${PO}_{4}^{3-}$ continued to decrease, but the decrease was small, indicating that the formation of hydration product precipitates was limited.

(9) $$nPAA+\frac{n}{2}{Mg}^{2+}\to nMg-PAA+nH^{+}$$

(10) $$nPAA+nK^{+}\to nK-PAA+nH^{+}$$

### 3.6. TG/DTG

Figure 7 shows the TG/DTG curves of MKPC suspensions at different hydration times: TG/DTG curves of #1 (a), (b); TG/DTG curves of #2 (c), (d); TG/DTG curves of #3 (e), (f). The red dashed area corresponds to the enlarged image. No thermal decomposition phenomenon of ${\mathrm{KH}}_{2}{\mathrm{PO}}_{4}$ was found in #1, #2, and #3 during the heating process, indicating that ${\mathrm{KH}}_{2}{\mathrm{PO}}_{4}$ reacted completely. With the progress of the hydration reaction, the content of MKPC hydration products gradually increased. The hydration products observed at 6 h were the same, all composed of ${\mathrm{Mg}}_{3}{\left({\mathrm{PO}}_{4}\right)}_{2}\cdot 22H_{2}O$ and ${\mathrm{MgKPO}}_{4}\cdot 6H_{2}O$. Moreover, with the increase of PAAS content, the content of MKPC hydration products decreased significantly, indicating that PAAS inhibited the formation of hydration products. A weight loss peak of Mg-PAA was observed in #2 and #3 at around 530 °C. According to the ICP analysis results, the slow increase of Mg^2+^ concentration was due to two aspects: on the one hand, the weakly alkaline pH environment of the suspension inhibited the dissolution of MgO; on the other hand, PAAS hydrolyzed to generate PAA (Equation (6)), which combined with free Mg^2+^ to form Mg-PAA (Equation (7)). With the progress of the hydration reaction, the weight loss peak of Mg-PAA gradually increased, indicating that the formation of insoluble Mg-PAA precipitate was a continuous process, leading to a decrease in the content of free Mg^2+^. Therefore, the content of hydration products in #3 MKPC was relatively low. The weight loss peaks of #3 MKPC at around 350 °C and 420 °C were the thermal decomposition of PAA and PAAS, respectively, indicating that PAAS was saturated in the #3 MKPC suspension and there was unhydrolyzed PAAS [45,46].

For the #1 suspension, two obvious weight loss peaks were observed at around 75 °C and 110 °C. Combined with XRD qualitative analysis (Figure 5a), the main hydration products were found to be ${\mathrm{MgKPO}}_{4}\cdot 6H_{2}O$ and ${\mathrm{Mg}}_{3}{\left({\mathrm{PO}}_{4}\right)}_{2}\cdot 22H_{2}O$ [47,48]. The weight loss peak at around 110 °C corresponded to ${\mathrm{MgKPO}}_{4}\cdot 6H_{2}O$, while the weight loss peak at around 75 °C was attributed to ${\mathrm{Mg}}_{3}{\left({\mathrm{PO}}_{4}\right)}_{2}\cdot 22H_{2}O$. At 10 min after the start of hydration, the main precipitate was ${\mathrm{MgKPO}}_{4}\cdot 6H_{2}O$, whose content gradually increased with the progress of hydration. The formation of ${\mathrm{Mg}}_{3}{\left({\mathrm{PO}}_{4}\right)}_{2}\cdot 22H_{2}O$ was observed at 8 min, which then gradually dissolved and converted into ${\mathrm{MgKPO}}_{4}\cdot 6H_{2}O$ until 12 min. After 12 min, the content of ${\mathrm{Mg}}_{3}{\left({\mathrm{PO}}_{4}\right)}_{2}\cdot 22H_{2}O$ continued to increase until the end of the hydration reaction. For the #2 and #3 suspensions, the weight loss peaks observed at around 110 °C at 6 min and 10 min were ${\mathrm{MgKPO}}_{4}\cdot 6H_{2}O$. With the progress of hydration, the weight loss peaks of ${\mathrm{Mg}}_{3}{\left({\mathrm{PO}}_{4}\right)}_{2}\cdot 22H_{2}O$ were observed at around 75 °C at 20 min and 18 min, respectively. The contents of both gradually increased with the hydration reaction, which was consistent with the XRD analysis results (Figure 5b,d,f). This explains why the conductivity of the #2 suspension (Figure 6b) increased before 10 min and then gradually decreased: before 10 min, less hydration product precipitate was formed in #2 suspension, resulting in reduced Mg^2+^ consumption, and the dissolution rate of MgO was greater than the precipitation rate of hydration products. After 10 min, Mg^2+^ formed hydration product precipitates and complexed with PAA to form Mg-PAA. For the #3 suspension, excessive PAAS was added, leading to the combination of PAA and Mg^2+^ to form Mg-PAA; thus, the conductivity continued to decrease.

### 3.7. FTIR

**Figure 8:**
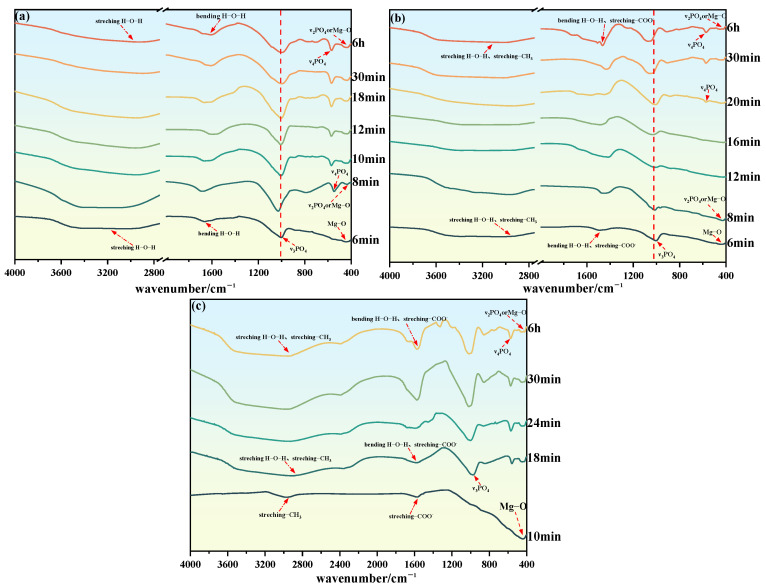
FTIR spectra of #1, #2, and #3 MKPC at different hydration times: (**a**) #1 MKPC; (**b**) #2 MKPC; (**c**) #3 MKPC.

Figure 8 shows the FTIR spectra of MKPC at different hydration times: (a) #1 MKPC; (b) #2 MKPC; (c) #3 MKPC. The red arrows correspond to different infrared characteristic peaks. For the #1 suspension, at 6 min of hydration, the H-O-H bending vibration peak and broad H-O-H stretching vibration peak of ${\mathrm{MgKPO}}_{4}\cdot 6H_{2}O$ were observed at wave numbers of 1657 cm^−1^, 2800 cm^−1^, and –3400 cm^−1^, indicating that free water gradually existed in the form of bound water with the progress of hydration reaction. The stretching vibration of ν_3_PO_4_ occurred at a wave number of 992 cm^−1^, and the Mg-O stretching vibration was observed at a wave number of approximately 438 cm^−1^, indicating that this stage was composed of ${\mathrm{MgKPO}}_{4}\cdot 6H_{2}O$ precipitate and undissolved MgO. At 8 min, a further ν_4_PO_4_ bending vibration was observed at the wave number of 548 cm^−1^, and the wave number at approximately 430 cm^−1^ may be caused by a ν_2_PO_4_ bending vibration or Mg-O stretching vibration. The shift of the broad H-O-H stretching vibration peak at 2800–3400 cm^−1^ to higher wave numbers indicates the transformation of hydration products during the hydration process. Combined with XRD qualitative analysis (Figure 5a), the hydration products at this stage were ${\mathrm{MgKPO}}_{4}\cdot 6H_{2}O$ and ${\mathrm{Mg}}_{3}{\left({\mathrm{PO}}_{4}\right)}_{2}\cdot 22H_{2}O$. The difference between the main infrared spectral absorption peaks of the two was small, so they could not be distinguished by FTIR. With the hydration proceeding to 10 min, the broad H-O-H stretching vibration peak at 2800 cm^−1^–3400 cm^−1^ shifted to a lower wave number until 12 min, indicating that ${\mathrm{Mg}}_{3}{\left({\mathrm{PO}}_{4}\right)}_{2}\cdot 22H_{2}O$ gradually dissolved and converted into ${\mathrm{MgKPO}}_{4}\cdot 6H_{2}O$. At 6 h, the FTIR characteristic peaks corresponded to ${\mathrm{MgKPO}}_{4}\cdot 6H_{2}O$ and ${\mathrm{Mg}}_{3}{\left({\mathrm{PO}}_{4}\right)}_{2}\cdot 22H_{2}O$, indicating that the final hydration products of #1 suspension were composed of ${\mathrm{MgKPO}}_{4}\cdot 6H_{2}O$ and ${\mathrm{Mg}}_{3}{\left({\mathrm{PO}}_{4}\right)}_{2}\cdot 22H_{2}O$.

For the #2 and #3 suspensions with different mass dosages of PAAS, the -CH_3_ stretching vibration peak and -COO^−^ asymmetric stretching vibration peak were observed at around wave numbers of 2900 cm^−1^ and 1600 cm^−1^, respectively, which overlapped with the broad H-O-H stretching vibration peak and H-O-H bending vibration peak. For the #2 suspension, at 6 min, the stretching vibration of ν_3_PO_4_ and Mg-O stretching vibration were observed at wave numbers of 996 cm^−1^ and 432 cm^−1^, respectively; in addition, the H-O-H bending vibration peak at a wave number of 1505 cm^−1^ and the broad H-O-H stretching vibration peak at 2900 cm^−1^–3400 cm^−1^ corresponded to ${\mathrm{MgKPO}}_{4}\cdot 6H_{2}O$. With hydration proceeding to 20 min, the ν_4_PO_4_ bending vibration was observed at a wave number of 570 cm^−1^, and the shift of the broad H-O-H stretching vibration peak at 2900 cm^−1^–3400 cm^−1^ to a higher wave number indicated a change in the type of hydration products. Combined with XRD qualitative analysis (Figure 5b), the formation of ${\mathrm{Mg}}_{3}{\left({\mathrm{PO}}_{4}\right)}_{2}\cdot 22H_{2}O$ was observed at this stage. For the #3 suspension, no infrared spectral absorption peak of hydration products was found at 10 min, and the Mg-O stretching vibration was observed at 433 cm^−1^, indicating that no hydration products were generated at this stage, and only MgO dissolution occurred. Therefore, the -COO^−^ asymmetric stretching vibration and -CH_3_ stretching vibration were observed at wave numbers of 1578 cm^−1^ and 2962 cm^−1^, respectively. At 18 min, the ν_2_PO_4_ bending vibration and ν_3_PO_4_ stretching vibration were observed at wave numbers of 432 cm^−1^ and 978 cm^−1^, respectively; in addition, the H-O-H bending vibration peak at a wave number of 1578 cm^−1^ and the broad H-O-H stretching vibration peak at 2800 cm^−1^–3400 cm^−1^ corresponded to ${\mathrm{MgKPO}}_{4}\cdot 6H_{2}O$, indicating the formation of ${\mathrm{MgKPO}}_{4}\cdot 6H_{2}O$. With the hydration process of the #2 and #3 suspensions proceeding to 6 h, their FTIR characteristic peaks were consistent with those of ${\mathrm{MgKPO}}_{4}\cdot 6H_{2}O$ and ${\mathrm{Mg}}_{3}{\left({\mathrm{PO}}_{4}\right)}_{2}\cdot 22H_{2}O$, indicating that the addition of PAAS did not change the composition of the final hydration products of MKPC.

### 3.8. Microstructure

**Figure 9:**
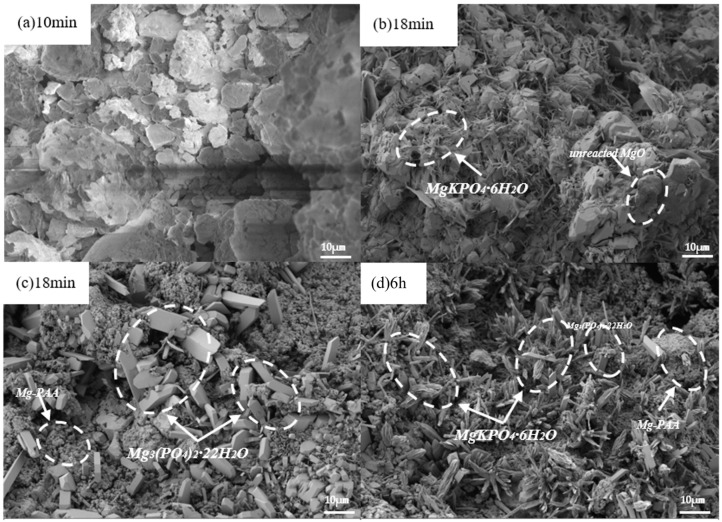
Microstructure of the #3 MKPC at different hydration times: (**a**) 10min, (**b**) 18min, (**c**) 18min, (**d**) 6h.

Figure 9 shows the microstructure of hydration products of #3 MKPC at hydration times of 10 min, 18 min, and 6 h. No obvious hydration products were found at 10 min, mainly undissolved dead-burned MgO particles, which exhibited irregular shapes, uneven sizes, rough surfaces, porous or layered structures, and tight stacking between particles. However, in some areas, there might be some gaps or pores between particles (Figure 9a). At 18 min, obvious hydration products were observed, mainly divided into two types: one was an acicular polycrystalline ${\mathrm{MgKPO}}_{4}\cdot 6H_{2}O$ structure with no obvious regular arrangement, and the other was an irregular rod-like bulk ${\mathrm{Mg}}_{3}{\left({\mathrm{PO}}_{4}\right)}_{2}\cdot 22H_{2}O$ structure. Unreacted dead-burned MgO was still observed; a large number of spherical particles were tightly stacked in Figure 9c, with no obvious gaps between particles, showing a relatively continuous structure, smooth surface and no obvious pores. The -COOH in PAAS could chemically react with Mg^2+^ on the surface of MgO to form chemical bonds and generate magnesium carboxylate complexes, which were Mg-PAA observed in TG/DTG analysis (Figure 7f). Through the diffusion and extension of molecular chains, multiple MgO particles were wrapped together to form a stable wrapping system, so the large number of stacked particles at 18 min were MgO agglomerates. With the hydration reaction proceeding to 6 h, the MKPC precipitates were composed of ${\mathrm{Mg}}_{3}{\left({\mathrm{PO}}_{4}\right)}_{2}\cdot 22H_{2}O$, ${\mathrm{MgKPO}}_{4}\cdot 6H_{2}O$, and Mg-PAA.

## 4. Discussions

(1) Hydration mechanism of #1 MKPC suspension without PAAS addition.

**Figure 10:**
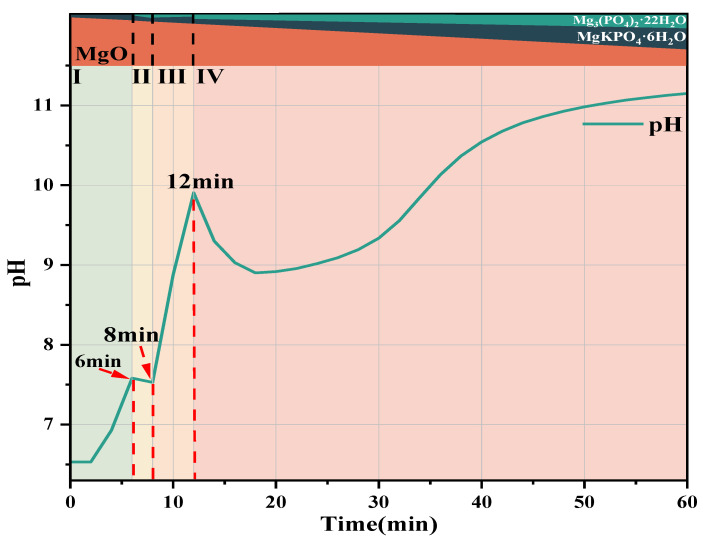
Schematic diagram of the development of hydration products of #4 MKPC without PAAS.

Based on the above experimental results, the schematic diagram of hydration product development of #1 MKPC without PAAS addition is shown in Figure 10, red line corresponds to the sample extraction time, which can be divided into the following four stages:

(1) First Stage (0–6 min): The first stage is mainly the complete dissolution of ${\mathrm{KH}}_{2}{\mathrm{PO}}_{4}$ to release hydrogen, potassium, and ${PO}_{4}^{3-}$ (Equations (3) and (4)), so the pH of the suspension is weakly acidic at 0 min. When dead-burned MgO is added, it combines with OH^−^ and partially dissolves to release Mg^2+^ and OH^−^ (Equation (6)), leading to a gradual increase in pH. Subsequently, free Mg^2+^, K^+^, and ${PO}_{4}^{3-}$ combine to form ${\mathrm{MgKPO}}_{4}\cdot 6H_{2}O$ and OH^−^ (Equation (9)), resulting in a further increase in the pH of the suspension while a slight decrease in conductivity due to the combined effect of ion generation and precipitation (Figure 5a).

(11) $$MgO+K^{+}+{HPO}_{4}^{2-}+{6H}_{2}O\to {MgKPO}_{4}\cdot {6H}_{2}O+{OH}^{-}$$

(2) Second Stage (6 min–8 min): The main characteristics of the second stage are the continuous formation of ${\mathrm{MgKPO}}_{4}\cdot 6H_{2}O$ (Equation (9)), and the combination of free Mg^2+^, ${PO}_{4}^{3-}$, and OH^−^ in the suspension to form ${\mathrm{Mg}}_{3}{\left({\mathrm{PO}}_{4}\right)}_{2}\cdot 22H_{2}O$ (Equation (10)). During this stage, through XRD quantitative analysis (Figure 5b) and ICP analysis, the decrease in ${PO}_{4}^{3-}$ concentration is greater than that in K^+^ concentration, indicating that ${\mathrm{Mg}}_{3}{\left({\mathrm{PO}}_{4}\right)}_{2}\cdot 22H_{2}O$ precipitation plays a dominant role, while the precipitation of ${\mathrm{MgKPO}}_{4}\cdot 6H_{2}O$ increases slowly. Therefore, the pH decreases from 7.58 to 7.52 during this stage, and the conductivity decreases nonlinearly (Figure 4a).

(12) $$3{Mg}^{2+}+2H_{2}{PO}_{4}^{-}+2{OH}^{-}+22H_{2}O\to {Mg}_{3}{\left({PO}_{4}\right)}_{2}\cdot 22H_{2}O+2H_{2}O$$

(3) Third Stage (8 min–12 min): The main feature of the third stage is that the ${\mathrm{Mg}}_{3}{\left({\mathrm{PO}}_{4}\right)}_{2}\cdot 22H_{2}O$ precipitate gradually dissolves to generate Mg^2+^, hydrogen ${PO}_{4}^{3-}$, and OH^−^ (Equation (11)). There are two sources of ${\mathrm{MgKPO}}_{4}\cdot 6H_{2}O$ in this stage: ① MgO, K^+^, ${PO}_{4}^{3-}$, and water in the suspension continuously form ${\mathrm{MgKPO}}_{4}\cdot 6H_{2}O$ and OH^−^ (Equation (9)). ② Mg^2+^ and hydrogen ${PO}_{4}^{3-}$ generated by the dissolution of ${\mathrm{Mg}}_{3}{\left({\mathrm{PO}}_{4}\right)}_{2}\cdot 22H_{2}O$ precipitate combine with K^+^ to form ${\mathrm{MgKPO}}_{4}\cdot 6H_{2}O$. Therefore, in the ICP analysis during this stage, the ${PO}_{4}^{3-}$ concentration increases while the $K^{+}$ concentration decreases. The dissolution and formation of the two hydration products simultaneously generate OH^−^, leading to a gradual increase in the pH of the suspension. At the end of this stage, part of the undissolved ${\mathrm{Mg}}_{3}{\left({\mathrm{PO}}_{4}\right)}_{2}\cdot 22H_{2}O$ precipitate still remains in the suspension.

(13) $${Mg}_{3}{\left({PO}_{4}\right)}_{2}\cdot 22H_{2}O+2H_{2}O\to 3{Mg}^{2+}+2H_{2}{PO}_{4}^{-}+2{OH}^{-}+22H_{2}O$$

(4) Fourth Stage (12 min-end of hydration): The main feature of the fourth stage is that ${\mathrm{Mg}}_{3}{\left({\mathrm{PO}}_{4}\right)}_{2}\cdot 22H_{2}O$ no longer dissolves. Its content gradually increases, as well as the continuous formation of ${\mathrm{MgKPO}}_{4}\cdot 6H_{2}O$ (Equation (9)). From 12 min to 18 min, the pH decreases from 9.9 to 8.9 because the precipitation of ${\mathrm{Mg}}_{3}{\left({\mathrm{PO}}_{4}\right)}_{2}\cdot 22H_{2}O$ consumes OH^−^ (Equation (11)), which plays a dominant role. With the progress of hydration, ${\mathrm{Mg}}_{3}{\left({\mathrm{PO}}_{4}\right)}_{2}\cdot 22H_{2}O$ gradually reaches saturation, and the precipitation rate of ${\mathrm{Mg}}_{3}{\left({\mathrm{PO}}_{4}\right)}_{2}\cdot 22H_{2}O$ slows down. Subsequently, the suspension continues to hydrate to continuously generate ${\mathrm{MgKPO}}_{4}\cdot 6H_{2}O$ and OH^−^ (Equation (9)), leading to a gradual increase in pH until the end of hydration.

(2) Hydration mechanism of #3 MKPC suspension with PAAS addition.

**Figure 11:**
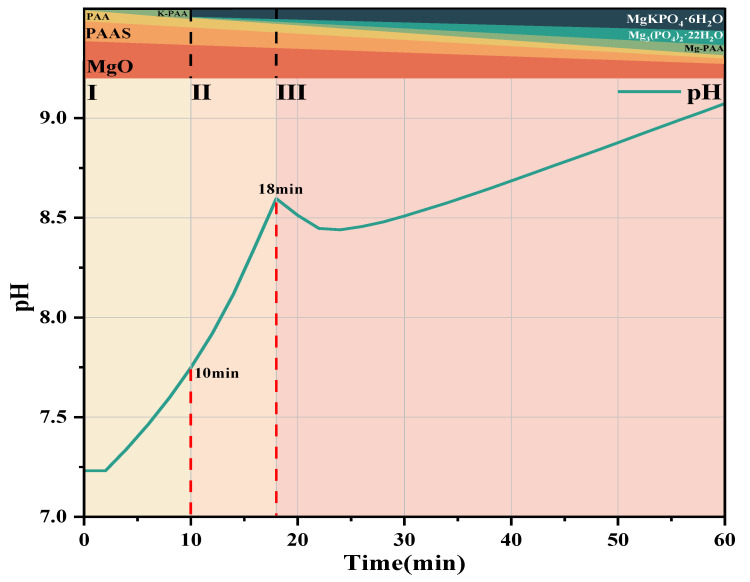
Schematic diagram of the development of hydration products of #6 MKPC with PAAS added.

Based on the above experimental results, the schematic diagram of hydration product development of #3 MKPC with PAAS addition is shown in Figure 11, red line corresponds to the sample extraction time, which can be divided into the following three stages:

(1) First Stage (0–10 min): No obvious hydration products were found in the first stage; the main processes were the complete dissolution of ${\mathrm{KH}}_{2}{\mathrm{PO}}_{4}$ (Equations (3) and (4)), the dissolution of dead-burned MgO (Equation (6)), and the hydrolysis reaction of PAAS (Equation (5)). No obvious hydration products were generated because the addition of PAAS increased the initial pH of the #3 MKPC suspension to a weakly alkaline level, resulting in low solubility of MgO. However, the dissolution of ${\mathrm{KH}}_{2}{\mathrm{PO}}_{4}$ was not affected by PAAS, and PAAS hydrolyzed to generate PAA (Equation (5)), which combined with K^+^ to form K-PAA and H^+^ (Equation (8)) and then dissolved. Therefore, the K^+^ concentration slightly decreased in the ICP analysis (Figure 6c). In this stage, the dead-burned MgO had low solubility and dissolved slowly, and PAAS hydrolyzed to release OH^−^, leading to an increase in pH.

(2) Second Stage (10 min–18 min): The pH in the second stage was affected by various factors. First, the dissolution of MgO generated OH^−^, leading to an increase in pH. PAAS hydrolyzed to generate PAA (Equation (5)), which combined with Mg^2+^ to form insoluble precipitate Mg-PAA and H^+^ (Equation (7)). Since the suspension was weakly alkaline, the solubility of MgO was low, so the generated contents of Mg-PAA and H^+^ were small, having little impact on pH. Meanwhile, ${\mathrm{MgKPO}}_{4}\cdot 6H_{2}O$ and a small amount of ${\mathrm{Mg}}_{3}{\left({\mathrm{PO}}_{4}\right)}_{2}\cdot 22H_{2}O$ were observed in the MKPC suspension. Among them, the precipitation of ${\mathrm{MgKPO}}_{4}\cdot 6H_{2}O$ generated OH^−^ (Equation (9)), while the formation of ${\mathrm{Mg}}_{3}{\left({\mathrm{PO}}_{4}\right)}_{2}\cdot 22H_{2}O$ consumed OH^−^ (Equation (11)). However, the content of ${\mathrm{Mg}}_{3}{\left({\mathrm{PO}}_{4}\right)}_{2}\cdot 22H_{2}O$ precipitate was small, leading to a further increase in pH.

(3) Third Stage (18 min-end of hydration): The main feature of the MKPC suspension in the third stage is the continuous precipitation of ${\mathrm{MgKPO}}_{4}\cdot 6H_{2}O$ and ${\mathrm{Mg}}_{3}{\left({\mathrm{PO}}_{4}\right)}_{2}\cdot 22H_{2}O$. From 18 min to 24 min, the pH decreased from 8.59 to 8.44 because the formation of ${\mathrm{Mg}}_{3}{\left({\mathrm{PO}}_{4}\right)}_{2}\cdot 22H_{2}O$ precipitate consumed OH^−^, which played a dominant role. This was also confirmed by the ICP analysis (Figure 6c), where the decrease in ${PO}_{4}^{3-}$ concentration was greater than that in K^+^ concentration. Subsequently, the precipitation of ${\mathrm{MgKPO}}_{4}\cdot 6H_{2}O$ generated OH^−^, which became the dominant factor, led to a gradual increase in pH until the end of hydration.

## 5. Conclusions

To minimize interference from excess water and better examine MKPC hydration, this study investigated the effect of PAAS on the setting time and fluidity of MKPC at a low water-to-solid ratio (W/S = 0.18). Furthermore, the retarding mechanism of PAAS on MKPC was studied using a high water-to-solid ratio (W/S = 10) via measurements of pH value and electrical conductivity, combined with characterizations including XRD, TG/DTG, and FTIR. The main conclusions are as follows:

1. The dosage of PAAS was positively correlated with the setting time of MKPC, and negatively correlated with its fluidity and compressive strength. When the PAAS dosage reached 16%, the setting time was significantly prolonged by 795.41%, and the compressive strength decreased sharply, or even to no strength. PAAS could reduce the total heat release and heat release rate of MKPC; in practical engineering applications, a PAAS dosage of 4–8% is the optimal range for performance balance.

2. PAAS did not participate in the hydration reaction of MKPC, nor did it change the composition of precipitates at the end of hydration (still MgKPO_4_·6H_2_O and Mg_3_(PO_4_)_2_·22H_2_O), but only inhibited the formation of hydration products.

3. PAAS delayed the hydration of MKPC through two pathways: first, it increased the pH value of the MKPC suspension to inhibit the dissolution of MgO, and second, it hydrolyzed in water to generate polyacrylic acid (PAA). PAA combined with K^+^ and Mg^2+^ to form soluble potassium polyacrylate (K-PAA) and insoluble precipitate magnesium polyacrylate (Mg-PAA), thereby reducing the amount of Mg^2+^ involved in the reaction; second, the carboxyl groups in PAAS chemically reacted with Mg^2+^ on the surface of MgO to generate the magnesium carboxylate complex Mg-PAA, enabling PAAS to be firmly adsorbed on the surface of MgO particles and wrap multiple MgO particles together. Moreover, this encapsulation system did not dissolve with the progress of hydration, but remained as a precipitate in the MKPC suspension. PAAS did not inhibit the dissolution of KH_2_PO_4_.

4. The core of MKPC hydration is the chemical process of MgO/KH_2_PO_4_ dissolution—ion complexation—precipitation of hydration products. PAAS regulates the reaction rate through chemical and physical effects without changing the nature of the reaction. A high water-to-solid ratio (W/S = 10) eliminates physical interference, clearly revealing the essence of chemical interactions, which still exists in low W/S systems and is the main reason for retardation.

5. Compared with conventional boron-based retarders, PAAS is the only retarder targeting MgO/Mg^2+^, which achieves synergistic retardation through multiple mechanisms. It has less interference from non-target ions in the system, is environmentally friendly, and does not affect the final hydration products of MKPC. Although it has performance side effects, it has greater potential for optimization, distinguishing it from traditional retarders with broad-spectrum effects, environmental risks, or product inhibition issues.

## Figures and Tables

**Table 1:** Chemical Composition of MgO/%.

Oxide	MgO	CaO	SiO_2_	Other
Content/%	≥90.42	≤1.51	≤3.16	≤0.79

**Table 2:** Mix Ratio Design for Low Water-to-Solid Ratio (W/S = 0.18).

NO.	MgO (g)	KH_2_PO_4_ (g)	H_2_O (g)	PAAS (g)	PAAS/(M+P) (%)
M-0	35.07	14.93	9	0	0
M-4	35.07	14.93	9	2	4
M-8	35.07	14.93	9	4	8
M-12	35.07	14.93	9	6	12
M-16	35.07	14.93	9	8	16

**Table 3:** Mix Ratio Design for High Water-to-Solid Ratio (W/S = 10).

NO.	MgO (g)	KH_2_PO_4_ (g)	H_2_O (g)	PAAS (g)	PAAS/H_2_O (%)
#1	35.07	14.93	500	0	0
#2	35.07	14.93	500	20	4%
#3	35.07	14.93	500	80	16%

## Data Availability

The original contributions presented in this study are included in the article. Further inquiries can be directed to the corresponding author.
